# Two methods of isolation of rat aortic smooth muscle cells with high yield

**DOI:** 10.1093/biomethods/bpae038

**Published:** 2024-05-22

**Authors:** Saran Lotfollahzadeh, Asha Jose, Esha Zarnaab Shafiq, Nourhan El Sherif, Michael Smith, Jingyan Han, Francesca Seta, Vipul Chitalia

**Affiliations:** Renal Section, Department of Medicine, Chobanian & Avedisian School of Medicine, Boston University, Boston, MA 02118, United States; Renal Section, Department of Medicine, Chobanian & Avedisian School of Medicine, Boston University, Boston, MA 02118, United States; Renal Section, Department of Medicine, Chobanian & Avedisian School of Medicine, Boston University, Boston, MA 02118, United States; Department of Biomedical Engineering, College of Engineering, Boston University, Boston, MA 02118, United States; Department of Biomedical Engineering, College of Engineering, Boston University, Boston, MA 02118, United States; Whitaker Cardiovascular Institute, Department of Medicine, Vascular Biology Section, Chobanian & Avedisian School of Medicine, Boston University, Boston, MA 02118, United States; Whitaker Cardiovascular Institute, Department of Medicine, Vascular Biology Section, Chobanian & Avedisian School of Medicine, Boston University, Boston, MA 02118, United States; Renal Section, Department of Medicine, Chobanian & Avedisian School of Medicine, Boston University, Boston, MA 02118, United States; Veterans Affairs Boston Healthcare System, Boston, MA 02118, United States; Center of Cross-Organ Vascular Pathology, Department of Medicine, Chobanian & Avedisian School of Medicine, Boston University, Boston, MA 02118, United States

**Keywords:** vascular smooth muscle cells, cardiovascular disease, thrombosis, cell survival, cell markers

## Abstract

Vascular smooth muscle cells (VSMCs) are an integral part of blood vessels and are the focus of intensive research in vascular biology, translational research, and cardiovascular diseases. Though immortalized vascular smooth muscle cell lines are available, their use is limited, underscoring the need for primary VSMCs. There are several methods for isolating primary cells from mice. However, the isolation method from rat blood vessels requires optimization, given the differences in the aorta of mice and rats. Here we compare two methods for VSMCs isolation from rats: enzymatic digestion and the “block” method. We observed a significantly higher yield of VSMCs using the enzymatic digestion method. We further confirmed that VSMCs expressed well-established VSMC-specific markers (calponin) with both methods and observed the persistence of this marker up to 9 passages, suggesting a continuation of the secretory phenotype of VSMCs. Overall, this work compares two methods and demonstrates a practical and effective method for isolating VSMCs from rat aorta, providing vascular biologists with a valuable and reliable experimental tool.

## Introduction

Vascular smooth muscle cells (VSMCs) are an integral component of the blood vessel wall. They modulate a plethora of biological functions and mechanical forces, thereby regulating vascular function [1]. Compared to other fully differentiated cells, VSMCs retain plasticity, a unique property by which VMSCs can “switch” between different functional states, i.e., a fully differentiated (contractile) state and a de-differentiated (proliferative/pro-inflammatory) state, in response to various stimuli, such as growth factors, cytokines, mechanic stress, or injury.^1^ Aberrant VSMC proliferation, migration, and extracellular matrix synthesis are implicated in forming the fibrous cap in atherosclerotic plaques, which can rupture and lead to thrombosis and infarction [2, 3]. Likewise, dysregulation of signaling pathways disrupts the differentiation state of VSMCs and consequently alters their functionality [4, 5], and contributes to the pathogenesis of arterial stiffness and calcification, aortic aneurysm, and thrombosis [6, 7]. Therefore, understanding the molecular mechanisms that regulate VSMC biology and function is critical for developing effective strategies for preventing and treating cardiovascular diseases.

Although immortalized VMSC cell lines are commercially available and widely used in research, they have several limitations, including an altered contractile phenotype, morphology, and metabolic function [8]; in some cases, these cell lines may even resemble other cell types, such as fibroblasts, macrophages or osteoblasts. The use of primary VSMCs became a desirable alternative.

Several methods have been described for isolating primary VSMCs from rodent models [8, 9]. However, they have limitations, including being labor-intensive, suboptimal yield, phenotypic switching, and being prone to fibroblast contamination [8, 9]. Furthermore, as most of the transgenic lines are of murine origin, it makes mouse VSMC isolation more common and desirable. Nonetheless, rats offer advantages in terms of higher VSMC yield due to their thicker aortic wall and a greater number of VSMC layers compared to mice [9]. Despite these advantages, effective protocols for isolating VSMCs from rats have been lacking. This knowledge gap is addressed in this study.

We set out to develop a reliable and simplified method for isolating VSMC from rat aortas. We utilized two distinct extraction methods to isolate primary VSMCs, ensuring their purity using multiple VSMC-specific markers, and demonstrating consistent morphological features over multiple passages. The optimized protocols for rat VSMC isolation can be leveraged by vascular biologists for deepening the understanding of smooth muscle cell biology and cardiovascular diseases.

## Materials

### Animal

A 12-week-old female Sprague Dawley rat, weighted at 275 grams, was purchased from Charles River Lab, strain code 400. The rat was housed individually in a cage and provided a rodent standard diet and water ad libitum. All procedures were performed in compliance with IACUC guidelines and under protocols approved by the Boston University Institutional Animal Care and Use Committee (IACUC).

### Rat aorta harvest

Surgical instruments:

1. Surgical drape
2. 1-ml syringe Gauge 27
3. CO_2_ incubator (Thermo Scientific Forma, cat. no. 370)
4. Ethanol gauze 2”x 2” sterile gauze
5. Petri dish
6. PBS (Gibco #100 10-023)
7. HBSS with calcium and magnesium (Gibco #14025092)
8. Collagenase 1 (3 mg/ml) (Sigma# SCR103)
9. Elastase (1 mg/ml) (Worthington-Biochem# LS002292)

### Immunostaining reagents

1. *Primary antibodies.*
  1. Alpha-smooth muscle actin (1A4/asm-1)–BSA-free—0.1 mg (Novus Bio-Catalog # NBP2-33006-0.1mg)
  2. Smooth muscle myosin (MYH11/923) (Novus Bio-Catalog # NBP2-44533-0.1 mg)
  3. Calponin 1 (D8L2T) XP^®^ Rabbit mAb #17819—100 µl (Cell Signaling, Catalog# 17819S)
2. *Secondary antibodies.*
  1. Goat anti-rabbit IgG Alexa Fluor 647 (Invitrogen: REF #A32733, LOT #WG324497)
  2. Goat anti-mouse IgG Alexa Fluor 594 (Invitrogen: REF #A32742, LOT #UJ293493)
  3. DAPI (Thermo scientific #62248)
  4. Coverslips
  5. Culture plates
  6. Dulbecco’s modified Eagle medium (DMEM) with 4.5 g/L glucose, 20% fetal bovine serum (FBS) and1% penicillin
  7. Conical flask shaker

## Methods

### Preparation and setup for rat aorta harvest

1. Prepare the hood workspace with sterile surgical tools. All surgical instruments are autoclaved, and the procedure is undertaken in sterile conditions.

#### Euthanasia

1. Euthanize the rats (N=3) with a continuous CO2 induction chamber.
2. Confirm the complete euthanasia with the absence of the toe pinch reflex.

### Aortic harvest

1. Shave the midline skin in the abdominal region and extend to half an inch bilaterally
2. Spray and wipe the shaved abdomen with 70% ethanol thrice, starting from the midline to the periphery in an eccentric manner
3. Transfer the fully euthanized, shaved animal to the laminar flow hood over ice immediately.
4. Perform a long midline laparotomy with straight surgical scissors, starting from the sub-xiphoid to the bladder.
5. Clamp the skin with hemostats bilaterally.
6. Exteriorize the intestines to the right abdominal side, and cover the intestine in a sterile 4x4 gauze.
7. Dissect the abdominal aorta away from the vena cava, starting from the subdiaphragmatic location to the iliac bifurcation, with iris scissors and atraumatic forceps.
8. Clamp the proximal and distal points of dissection.
9. Cut the dissected aorta between clamps, distal to the proximal clamp and proximal to the distal clamp.
10. Place the cut aorta in a sterile 100mm Petri dish containing 10 ml of cold PBS buffer.
11. To drain the remaining blood, the aorta is gently irrigated with fresh PBS into the lumen with a 1 mL sterilized syringe; the amount of PBS is adjusted until blood is no longer present inside the aorta. The aorta will appear translucent when blood has been drained Properly and completely.
12. Cut the aorta longitudinally.
13. Gently scrape off the endothelium with fine-toothed forceps or a scalpel.

### Isolating VSMCs

1. The aorta is divided in two halves, for both digestion and block methods.
2. One half is minced with fine micro-dissecting scalpels into less than 1-mm pieces inside a clean Petri dish containing 5–7 ml PBS (digestion method).
*Note: This isolation procedure should be performed gently to minimize the potential damage to SMCs; blunt performance with a scalpel will lower cell viability.*
3. One-half of the tissue is diced in about 1–2-mm-sized segments. These tissue segments will be transferred to a clean Petri dish containing 5–7 ml of PBS (block method).
*Note: The aortic isolation, adventitial ablation, and mincing of the aorta were all undertaken over the ice. All the steps after this process were conducted at 37ºC or room temperature. This strategy was adopted to reduce freeze and thaw cycles.*

In the following sections, the steps of both methods are distinguished by the prefix of A for digestion and B for block method.

#### A—Digestion method

A-20. Using sterile forceps, the adventitia-free, minced aorta is gently transferred to a 15-ml conical tube containing 5 ml of HBSS with calcium and magnesium, 15 mg of collagenase (3 mg/ml), and 5 mg of elastase (1 mg/ml).

*Note: If the aorta is not transferred immediately, it must be placed on ice for a maximum of 20 minutes.*

A-21: The minced aorta is digested for 45 minutes in a conical shaker flask with constant stirring (150–200 rpm) at 37°C.

*Note: This can be adjusted between 5 and 15 minutes, depending on the size and length of the aorta being minced.*

A-22: Every 15 minutes, utilizing a sterile wide bore pipette, pipette up and down gently to fully dissociate the tissue.

A-23: After 45 minutes, there should be little to no tissue clumps or minced tissue visible; in case clumps are still visible, increase digestion time for an additional 5–15 minutes. At the end of the digestion time, the solution should appear cloudy.

A-24: Centrifuge the solution at 1000 rpm for 5 minutes.

*Note: Do not spin longer or at higher speeds as cells will be damaged.*

A-25: Remove supernatant. Wash the cell pellet once by gently resuspending cells with 5 ml of cold PBS (to completely remove the digestion solution).

A-26: Centrifuge again at 1000 rpm for 5 minutes to remove the washing solution.

A-27: Remove supernatant and add 5 ml of DMEM with 4.5 g/L glucose, 20% FBS, and 1% penicillin.

A-28: Equally distribute the total volume (5 ml) among two 35-mm rat tail collagen-coated plates (2.5 ml each max) (Corning, NY, USA).

A-29: Store plates in a 37°C, 5% CO_2_ incubator.

A-30: After 24 hours, without disturbing cells and with minimal plate movement, add 0.5 ml of DMEM with 4.5 g/L Glucose, with 20% FBS and 1% of penicillin to each dish, without removing the medium.

A-31: Leave cells in the incubator for an additional 48 hours without any additions or movements (do not disturb cells for 48 hrs).

A-32: After 48 to 72 hours, the culture medium can be replenished with fresh medium.

#### B—Block method

B-20: Sterilize 12-mm round coverslips with ethanol (70%) and then UV light for 15 minutes in a culture hood.

B-21: Place sterilized coverslip in 35-mm plates.

B-22: Seed the aorta segments in one or two 35-mm plates.

B-23: Using sterile forceps, place the aortic segments, equally distant, in a row on the coverslip inside the culture dish with the intima side facing down for adherence and growth.

B-24: Carefully add 2 ml of DMEM with 4.5 g/L glucose, 20% FBS, and 1% penicillin without moving the tissue pieces; the medium should cover the tissue completely.

B-25: After 24 hours, without disturbing the aorta pieces and with minimal movement, add 0.5 ml of DMEM with 4.5 g/L glucose, 20% FBS and 1% penicillin to each dish.

*Note: Do not remove the old medium.* After the initial 72 hours, the culture medium is replenished with fresh medium

### Passaging cells

1. Aspirate out old media from cell plates
2. Wash with PBS
  1. Wash twice with 1 mL of PBS for 1–2 minutes. Aspirate out PBS.
  2. For the plate with a coverslip, ensure the coverslip is fully soaked under PBS. Then gently swirl the plate in a circular motion to detach the cover slip.

### Trypsinization

1. Add 0.5 ml of 0.25% trypsin to cover the bottom of the cell plate.
2. Incubate at 37°C for 3–4 minutes.
*Note: For a plate with a coverslip, ensure trypsin penetrates under the coverslip to detach all cells.*

B-29: Swirl and gently tap the sides of the plate to break up clusters of cells.

B-30: Check under the microscope whether most cells have detached and separated. Continue to gently tap plates if cells are still adherent or clustered.

B-31: Neutralize trypsin

B-31.1. Add 2 mL of full media (high glucose DMEM, 20% FBS, and 1% penicillin) into the cell plate to neutralize trypsin.

B-31.2. Gently pipette cell suspension up and down, making sure to wash the edges of the plate to minimize cell loss.

B-31.3. Pipette cell suspension from the plate into a 15-mL conical tube.

B-31.4. Add 1 mL of media into the cell plate and pipette up and down gently again, focusing on the edges of the plate.

B-31.5. If needed, use a cell scraper tool to scrape the edges of the plate, ensuring no cells remain on the plate.

B-31.6. Add the cell suspension to the same 15 mL conical tube and add media into the conical tube to reach a volume of 3 mL.

B-32. Seeding cells

B-32.1. Pipette 1.5 mL of the cell suspension into the new P60 plates.

B-32.2. Add 4 mL of media into P60 plates.

B-32.3. Place cell plates in the incubator at 37°C and 5% CO_2_ and let cells adhere overnight. Replace cell media every 48 hours.

### Freezing cells

1. Pipette out media by tilting the plate to ensure media is removed.
2. Follow steps 28–31.
3. Centrifuge the cell suspension at 1200 rpm for 8 minutes.
4. Remove supernatant.
  (Optional) Cell pellets can be washed 1–2 times with PBS to remove the medium completely.
5. Add 1 ml of freezing medium to the cell pellet and gently resuspend cells.
  1. Freezing medium: 10% DMSO in 90% FBS
  2. 1 ml of freezing medium per cryovial.

## Results

**Figure 1.**
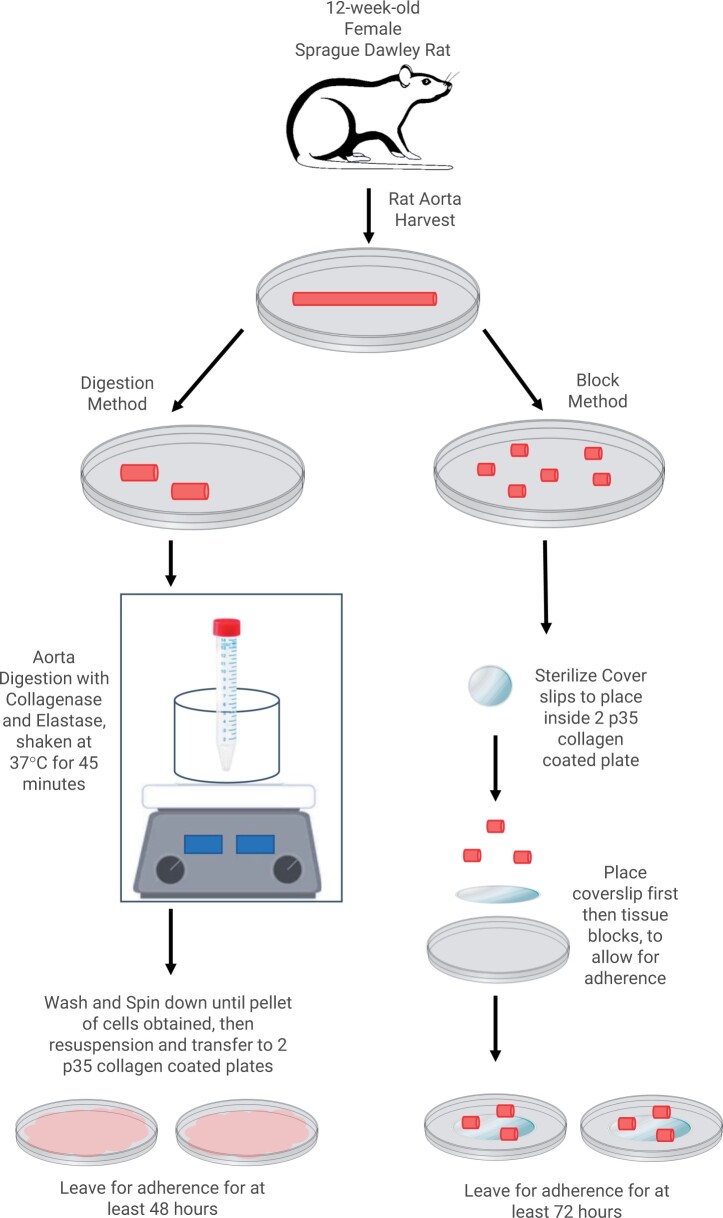
Experimental strategy. Experimental setup schematic of aorta harvest and sectioning for both digestion and block method

The overall procedure for VSMC isolation from rat aorta is illustrated in Fig. 1. An aorta harvested from a 12-week-old female Sprague Dawley rat was separated into two segments for the digestion and the block method, respectively. In the digestion method, before incubating the aortic tissue in a collagenase and elastase solution for 45 minutes at 37°C, the adventitia was stripped off and endothelium gently scraped off, Then the cells were spun, and the cell pellet was plated on collagen-coated plates. In the block method, segments of the aorta, about 1–2 mm in size, were covered with sterile coverslips to ensure the outgrowth of VSMCs from the aortic segment over time.

**Figure 2.**
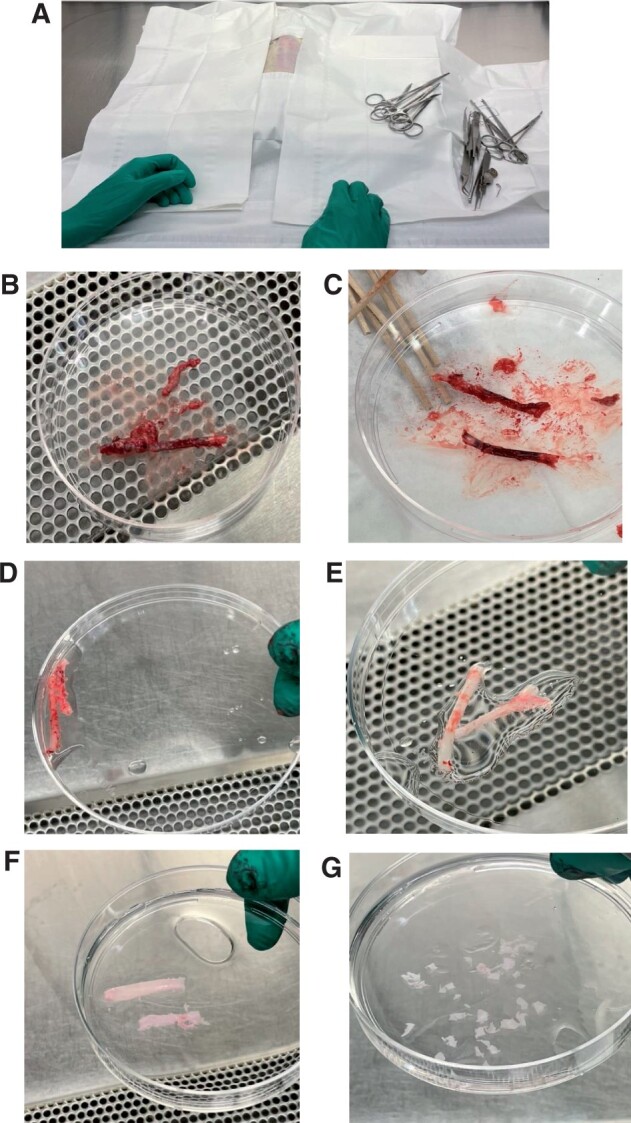
Aorta extraction from 12-week-old female Sprague Dawley rat. Notes: **(A)** Orientation of rat depicts complete sterilization of all surgical tools and sterile draped surgical bench. Rat is in the supine position, with exposure to the ventral abdomen exposed for incision. **(B)** The Rat aorta has been extracted by peeling it gently away from the spinal column. Aorta currently has excess tissue. **(C)** Excess tissue is removed, leaving stem-like aorta segments, in this case, the aorta was sectioned into two pieces to clean thoroughly. **(D)** Cleaned aorta segments are washed with PBS. (**E)** Fresh PBS has been injected into the lumen with a 1 mL sterilized syringe to drain blood. As blood is draining aorta will appear more translucent. The adventitial layer has been scraped off. **(F)** Adventitia and the outer portion of the media are completely detached. The aorta is cut open longitudinally and endothelium is scrapped off. **(G)** The aorta is cut into 1mm cubes, half are distributed into a new petri dish containing PBS, for digestion, and six pieces are maintained for the block method for cell culture.

The surgical procedure depicted in Fig. 2 illustrates the steps for both methods. The aorta was carefully dissected from perivascular connective tissue and fat; adventitia and endothelium were removed to minimize potential contamination of other cell types. Adventitia was removed with atraumatic forceps held perpendicular to the aortic long axis to ensure complete tissue removal without compromising the vessel integrity (**B, C, D, E**). Two meticulous attempts were performed. Panels E and F are representative of the procedure before and after adventitia removal. The cleaned aortic media was separated into two methods for observation (**F** and **G)**.

**Figure 3.**
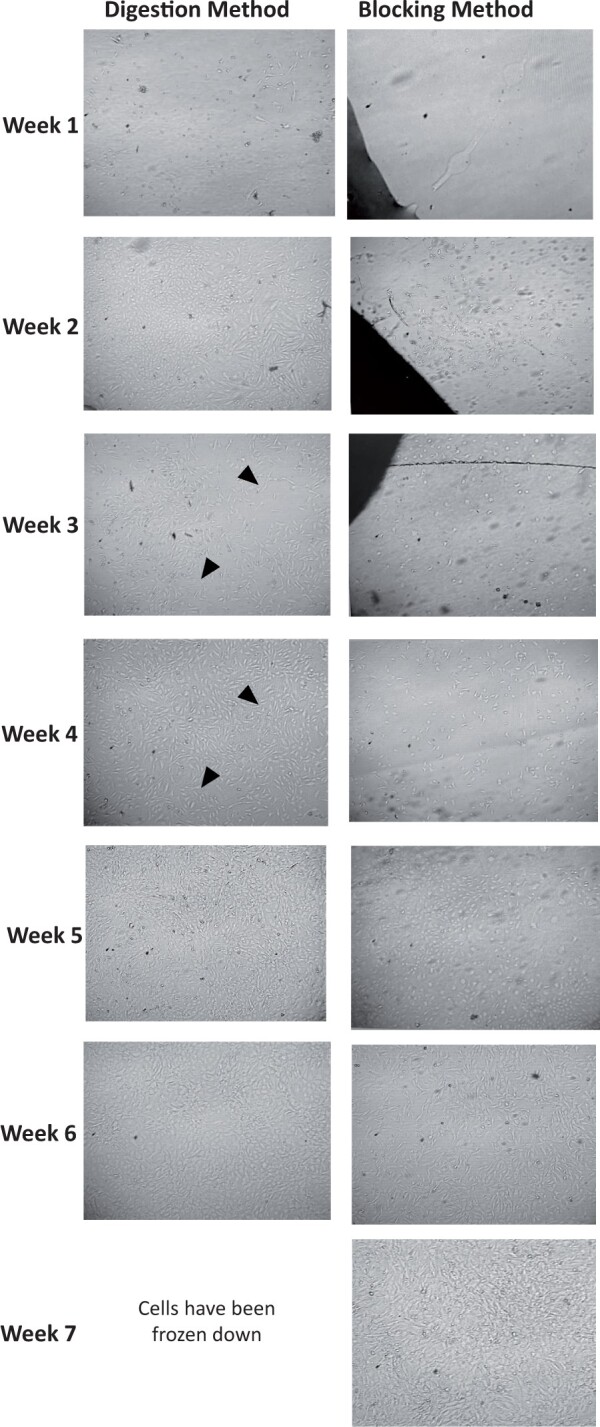
Comparison of cell growth between digestion and block method. Notes: In **Week 1** (after the initial 72 hours, images were taken), the cells obtained using the digestion method depict small, short-rounded and truncated T shape, while the block method portrays cells budding from the tissue block (left corner of image). Over **Weeks 2 and 3,** the cells isolated with the digestion method have elongated and are increasingly becoming more confluent, and the claw and T-shaped cells have contacted each other and are becoming sub-confluent. **In Week 3** in the block method, the cells increase, mainly budding along the tissue blocks, and adhering to the coverslip. The circular shape of the VSMCs is maintained. By **Week 4 in** the digestion method, the VSMCs have fully taken on the VSMC's polarity characteristics and are collectively making spindles. The proximity of the cells has increased, and the spindle shape is more uniform than in **Week 3.** **Week 4** in the block method indicated an increase in T-shaped cells, as observed in the digestion method in **Week 2.** In **Weeks 5 and 6 in** the digestion method, cells were cultured until they reached full confluency of about 80%–90% to ensure minimal loss of cells during the freezing and splitting process. Cells have abundant spindle shapes and are homogenous in polar bundles. The VSMCs characteristic of “hills and valleys’ has fully developed. In **Weeks 5 and 6** of the block method, cells transition from a plump T cell shape to an elongated spindle, similar to the digested cells in **Week 4.** In **Week 7**, the VSMCs in the block method reached 90% confluence and exhibited mature morphology; VSMCs are interlaced within each other to form a confluent monolayer.

The cells obtained from the two methods were cultured up to seven passages under identical conditions for comparison (Fig. 3). Cell morphology and confluency were compared throughout this period by acquiring weekly images (Fig. 3). From Week 1 onwards, spindle-shaped cells, typical of VSMC morphology, were observed with both methods. However, the number of cells and the extent of confluency were higher in the digestion method compared to the block method. Cells took ∼ 10 days to grow from coverslips in the block method. Over the following 2–3 weeks, more cells exhibited spindle shape, especially those in the confluent patches.

**Figure 4.**
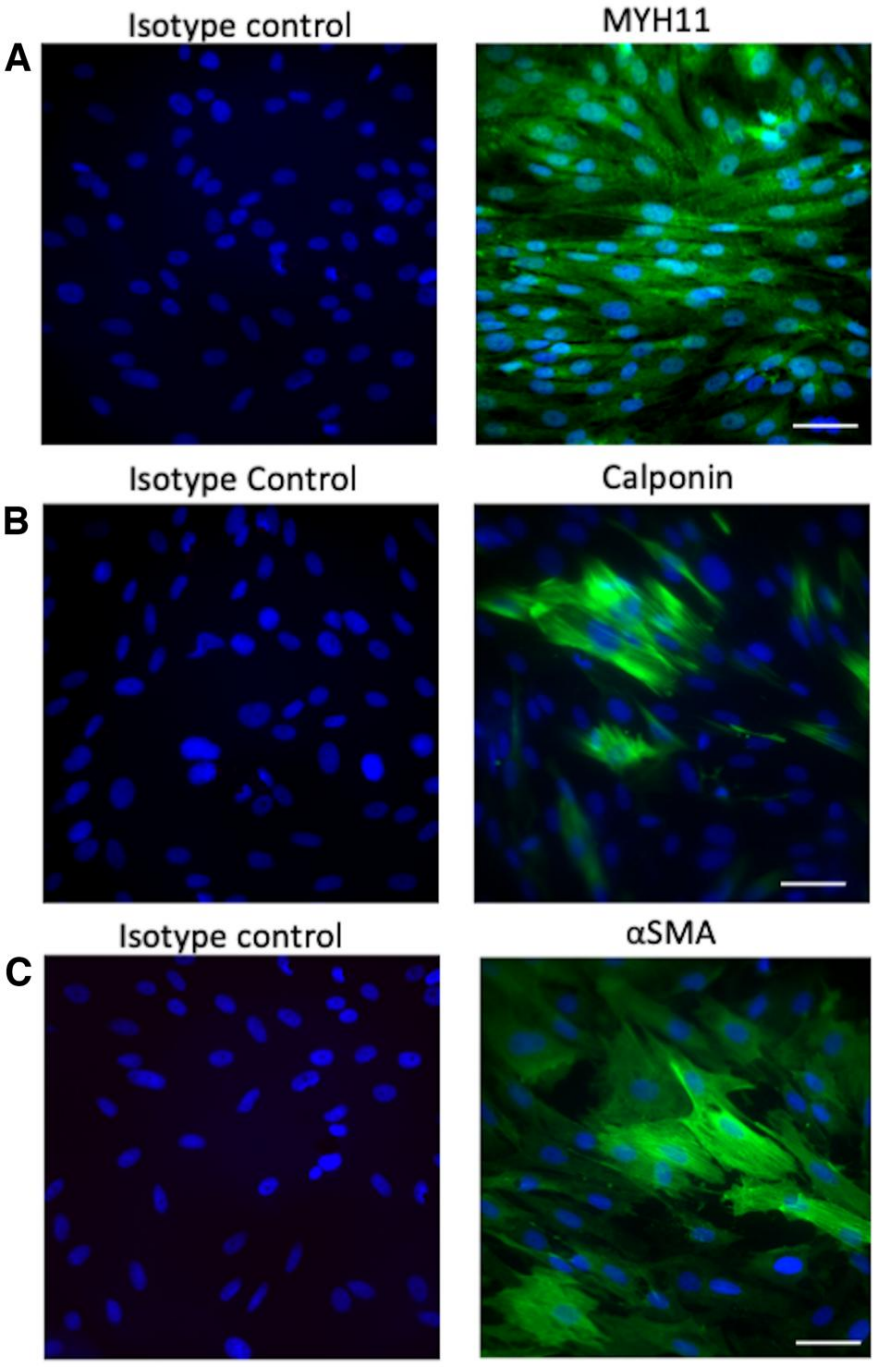
Formalin-fixed paraffin-embedded cut slides were stained with. Notes: **(A)** Calponin 1 (D8L2T) XP^®^ Rabbit mAb #17819 (Cell signaling #17819S) **(B)** Myosin heavy chain 11 Antibody (MYH11/923) (Novus Bio #NBP2-44533-0.1mg) **(C)** Alpha-Smooth Muscle Actin Antibody (1A4/asm-1)—BSA Free—(Novus Bio #NBP2-33006-0.1mg). Secondary antibodies **(A)** Goat anti-rabbit IgG Alexa Fluor 647 (Invitrogen: REF #A32733, LOT #WG324497) **(B) and (C)** Goat anti-mouse IgG Alexa Fluor 594 (Invitrogen: REF #A32742, LOT #UJ293493). DAPI was utilized as a nuclear stain. The images are presented with a high power field at Scale bar = 50 microns

To further characterize the isolated VSMCs and check their purity, we stained cell lysates of VSMCs with well-established VSMC markers: calponin 1, smooth muscle myosin, and α-smooth muscle actin (Fig. 4) [11, 12]. These markers are commonly utilized to distinguish VSMCs from fibroblasts and other cell types that may be present due to contamination from the intima or adventitia.

Calponin 1 (gene: CNN1) is an actin filament regulatory protein commonly expressed in smooth muscle cells.^13^ Specifically, calponin 1 is a cytosolic protein known to regulate the smooth muscle myofilaments and enhance contractility and smooth muscle cell proliferation.^13^ Smooth muscle myosin heavy chain (gene name: MYH11) is a VSMC-specific contractile protein and is expressed in VSMC with halted proliferation and migration activity.^12^ α-smooth muscle actin (SMA; gene name: ACTA2), is a contractile/cytoskeletal protein that, together with smooth muscle myosin, contributes to VSM-generated mechanical tension (actin-myosin crossbridge).^14^ SMA is normally restricted to VSMCs, but it can also be expressed in certain non-muscle cells, most notably myofibroblasts.^14^

Immunofluorescence staining showed that >95% of cells expressed smooth muscle myosin, confirming the VSMCs contractile phenotype and high purity of our VSMC preparation. CNN1 and SMA were observed at lower levels than smooth muscle myosin. There was no difference in the expression of markers between the two extraction methods. Overall, these results suggest that VSMCs with contractile phenotype were successfully obtained with both methods.

**Figure 5.**
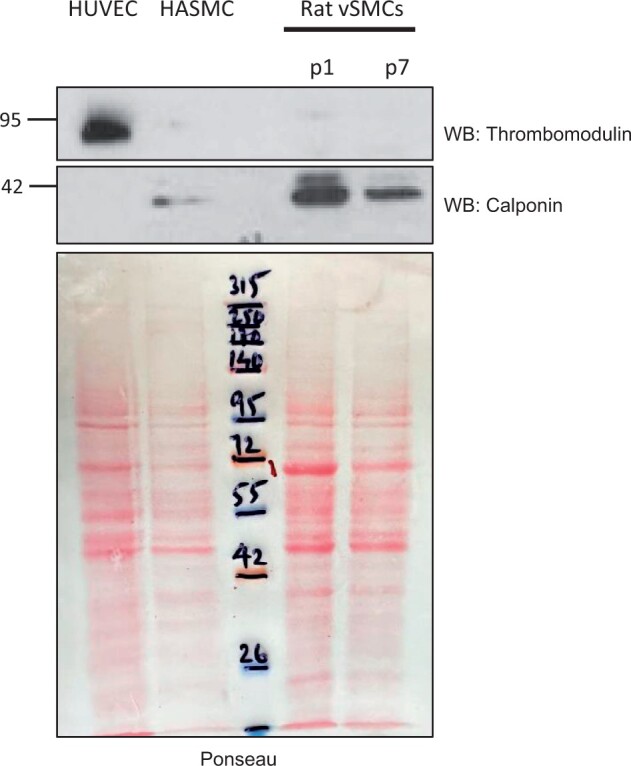
VSMCs were isolated from the rat aorta and continued till passage 7, harvested and probed for thrombomodulin, an endothelial marker and a VSMC marker, calponin 1. As a positive control, we used primary human VSMCs (HASMC) which express calponin 1. Primary HUVECs were used, as negative control for VSMCs, which highly expressed thrombomodulin. Ponceau red was used as a loading control

We further examined the contamination of VSMCs with endothelial cells. Calponin is a specific marker of VSMCs. The primary vascular smooth muscles from the rat aorta continued until passage 7. Passages 1 and 7 VSMCs were harvested and probed for thrombomodulin and calponin. We used primary human umbilical vein endothelial cells (HUVECs) and primary human VSMCs as positive controls. Ponceau served as a loading control. Figure 5 shows that calponin was expressed in primary human VSMCs and persisted until passage 7 in rat VSMCs. Thrombomodulin is expressed in HUVECs. However, it was distinctly absent from human or rat VSMCs. The above data suggested a lack of contamination of endothelial cells and the presence of rat VSMCs in our culture. The VSMC features persisted for seven passages. Calponin is a marker of secretory VSMCs.

## Discussion

The current protocol describes a direct comparison between two methods to isolate VSMCs from aorta. This approach is easy and user-friendly primary rat VSMCs. Both the adventitia and intima were removed to reduce the contamination by different cell types and to ensure optimum VSMC purity. VSMCs in our culture conditions continued to display the VSMC markers for up to seven passages. The pros and cons of both the methods are described in Table 1. However, in our hands, the enzymatic method yielded higher number of VSMCs. While both methods are easy and reproducible, the digestion method is easier to adopt. The digestion method requires the pieces of the aorta to be treated with a pre-defined concentration of enzymes. Small pieces of aorta allow penetration of enzymes in the wall. This is a protocolized method, which improves its adaptability by relatively untrained personnel. Our team comprises experienced postdoctoral fellows and graduate and undergraduate students. Undergraduate and graduate students took two attempts to familiarize themselves with the methods.

**Table 1.** Advantages, disadvantages, and duration of digestion and block methods used to isolate the rat VSMC.

	Digestion method	Block method
Advantages	Higher VSMC yield (due to thicker aortic wall/greater number of VSM layers compared to mice) Higher number of cells Higher extent of confluence	Higher VSMC yield (due to thicker aortic wall/greater number of VSM layers compared to mice) Simpler protocol compared to digestion method (does not require further steps for digestion)
Disadvantages	More complex protocol compared to block method (requires additional steps for digestion)	Lower number of cells Lower extent of confluence
Duration	Aorta harvest—15 minutes Digestion—45 minutes Washing—10 minutes Seeding—5 minutes Total = 75 minutes	Aorta harvest—15 minutes Sterilization—15 minutes Seeding—5 minutes Total = 35 minutes

Conventional VSMC isolation methods from rats have several disadvantages, including low yield, contamination with other cell types, and phenotype switching of VSMCs [10–13]. These issues in part arise from limited dissection that only removes the intimal layer, leaving the adventitia rich in pericytes and fibroblasts, which can contaminate VSMCs. Moreover, the proportion of fibroblasts can increase with subsequent passaging because they replicate more rapidly than VSMCs. Therefore, removing adventitia and intima proved essential to reduce endothelial and fibroblast contamination and improve the yield and purity of VSMC. Adventitia was removed with atraumatic forceps held perpendicular to the aortic long axis to ensure the complete removal of the tissue without compromising the vessel integrity.

We performed additional validation of the isolated cells from the rat aorta with immunostaining for VSMC-specific markers [11, 12]. It is interesting to note that all the VSMCs expressed smooth muscle myosin, while 50% of VSMCs expressed calponin 1. A small fraction of myosin-expressing cells were positive for αSMA. These differences in levels of VSMC markers can potentially be explained by the expression of different contractile proteins at different time points during the VSMC progression in cell culture.

The methods described in the literature for the isolation of rat VSMC are compared against our methods in Table 2. Out of all the methods, our work demonstrates the ease of the enzymatic method. We have applied only to macro vasculature. However, it is possible to adopt this method to digest and extract endothelial cells from microvasculature. The time and concentration of the enzyme will change and have to be optimized for the microvascular bed depending on various parameters, such as the extracellular matrix and the delicacy of the endothelial cells. The block method cannot be adopted for microvasculature as cutting the soft tissue with microvasculature without damaging it will be difficult.

**Table 2:** Pros and cons of isolation methods of VSMC from available literature.

Study	Advantages	Disadvantages
**Jin *et al.* (2021)** [ 14 ]	Uniform and precise sizing of 75 uM or less for the processed aortic segments	The study did not confirm purity of VSMCs by staining for cell markers Visual observation cannot differentiate between fibroblasts and VSMCs, especially after phenotypic switching or maturing of the VSMCs The study did not assess contractile phenotypic characteristics Lacking in a comprehensive description of the digestion process This method used a grinding technique, which could potentially damage the cells
**Tai *et al* . (2008)** [ 15 ]	Mechanical endothelial dissection Probing the absence of endothelial cell contamination with the endothelial cell markers, (eNOS, von Willebrand factor) Confirming the absence of endothelial cells in the culture by assessing the expression of eNOS mRNA by RT-PCR analysis	Lack of interval probing the cell proliferation, not until the 5th day after removal of aortic pieces from the growing cells on the coverslips (Blocking Method) that could potentially provide a more consecutive course of cell proliferation and fate Adventitia layer was not removed leading to an increased possibility of pericytes and fibroblast contamination cells. Stripping off adventitia allows for the purest form of non-contaminated VSMCs as defined in our study Method did not remove the fat layer of the aorta leading to an increased possibility of endothelial and adipocyte cell contamination Incomplete characterization of cells due to lack of staining for cell markers
**Kizub *et al* . (2010)** [ 16 ]	Single smooth muscle cells were dispersed from the rat thoracic aorta enzymatically	Adventitia layer was not removed leading to an increased possibility of pericytes and fibroblast contamination cells. Stripping off adventitia allows for the purest form of non-contaminated VSMCs as defined in our study Intima was not scraped leading to an increased risk of endothelial contamination Papain enzyme was used, which can non-specifically damage the contractile tissue thereby compromising the function of VSMCs
**Sun *et al* . (2021)** [ 17 ]	Describing two separate tissue explants method and the enzyme digestion method The removal efficiency of the intima and adventitia was confirmed by hematoxylin–eosin and immunohistochemical staining. The method was observed to exhibit fibroblast contamination after conducting immunohistochemical staining for α-smooth muscle actin (α-SMA)	Insufficient information available regarding the digestion process and the characterization Examination of cellular morphology revealed endothelial cell contamination in the method employed
**Ruef *et al* . (1998)** [ 18 ]	Rat aortic smooth muscle cell were isolated from the thoracic aortas of Sprague-Dawley rats by enzymatic digestion. Three different isolates were used	Insufficient information available regarding the digestion process and the characterization
**Weber *et al*. (2011)**[19]	VSMCs are isolated from fetal rat ductus arteriosus of high purity and viability	Requires extensive purification, including using magnetic column to isolate cells There is a likelihood of loss of cells and viability through purification and suspension methods used Costly added steps of purification, and purification mediums, as well as filtration columns, and cell sorting Used gelatin coated flasks to grow cells. In our study, the lack of collagen coated plates may reduce the proliferation and migration of VSMCs out of the aortic tissue in the first passage

This study has limitations. Here, we performed the isolation protocol on only female rats. Male rats are likely to have thicker aortas, which may improve the yield of VMSCs. We isolated rat VSMCs from healthy rats. Further work is needed to validate this protocol in rats with different disease models and perform functional assays to characterize them better. HUVECs were used as a model of endothelial cells, which is not the most appropriate model for arterial ECs. Although thrombomodulin is used as an endothelial marker, it is also expressed in other non-vascular cell types.

In conclusion, the digestion and block methods yielded viable VSMCs. However, the digestion method produced more VSMCs than the block method. Our study provides a reliable method for extracting primary rat VSMCs, with greater yield and purity, as well as no phenotype switching up to seven passages in culture.

## Data Availability

The data underlying this article are available upon request to the corrospinding author with propoer regulatory approvals. *Conflict of interest statement*. The authors have declared that no conflict of interest exists.
